# Community Interactions and Extracellular Riboflavin Are Associated with Oral Biofilm-Mediated Medical Stainless Steel Corrosion

**DOI:** 10.3390/microorganisms14091938

**Published:** 2026-09-02

**Authors:** Siyang Dai, Weihao Lan, Weijia Geng, Pan Liu, Bujian Wang, Xun Li, Yongqiang Fan, Fuhui Wang, Dake Xu, Ying Zheng

**Affiliations:** 1 College of Life and Health Sciences, Northeastern University, Shenyang 110819, China; 2 State Key Laboratory of Digital Steel, School of Materials Science and Engineering, Northeastern University, Shenyang 110819, China; 3 Key Laboratory for Anisotropy and Texture of Materials (Ministry of Education), School of Materials Science and Engineering, Northeastern University, Shenyang 110819, China; 4 Foshan Graduate School of Innovation, Northeastern University, Foshan 528311, China; 5 Liaoning Province Key Laboratory of Oral Disease, Shenyang Clinical Medical Research Center of Orthodontic Disease, The First Clinic, Orthodontic Department, School and Hospital of Stomatology, China Medical University, Shenyang 110001, China

**Keywords:** oral microbiota, microbiologically influenced corrosion, interspecies interactions, riboflavin

## Abstract

Microbiologically influenced corrosion (MIC) at oral biomaterial interfaces is viewed as an ecological phenomenon, yet how microbial composition and interspecies interactions shape corrosion remains unclear. Here, we investigated whether oral microbial community composition and interspecies interactions contribute to medical 316L stainless steel corrosion. Consortia-enhanced Cr and Fe release and localized surface pitting, with marked inter-subject variability. Interface-associated biofilms exhibited trends toward compositional shifts and showed enrichment of predicted pathways for fermentation and riboflavin metabolism, along with higher genome-based metabolic interaction potential than planktonic communities. Extracellular riboflavin accumulated in MIC systems and correlated positively with dissolved Cr and Fe concentrations. In perturbation assays, riboflavin supplementation increased corrosion current density (*i*_corr_) and metal dissolution, whereas roseoflavin reduced extracellular riboflavin availability and corrosion-related parameters without marked changes in the measured biofilm biomass or surface-associated ATP levels. A defined three-strain consortium (*C. tsuruhatensis*, *R. erythropolis*, and *T. aromatica*) reconstituted the S3 high-corrosion phenotype, including elevated *i*_corr_, extracellular riboflavin accumulation, and induced pitting, consistent with a proposed riboflavin-linked model involving species-dependent metabolic interactions. These findings suggest that extracellular riboflavin may represent a candidate redox-active factor associated with microbial community interactions and corrosion activity, providing an ecological framework for understanding microbiota-associated corrosion resistance at oral biomaterial interfaces.

## 1. Introduction

Microbial communities colonizing solid surfaces can generate physicochemical microenvironments that differ markedly from the surrounding bulk phase [1,2,3]. Within multispecies biofilms, spatial organization, nutrient exchange, competition, and metabolite retention shape local pH, oxygen availability, redox potential, and interfacial chemistry [4,5]. Consequently, functions expressed at a colonized surface may be emergent properties of community assembly rather than predictable consequences of the abundance or activity of individual taxa alone [6]. Understanding how such community-level properties arise is particularly important at biomaterial interfaces, where microbial biofilms can modify the stability of the underlying material.

The oral cavity provides a dynamic and clinically relevant system in which to investigate these processes [7,8,9]. Oral microorganisms colonize teeth, mucosal surfaces, and artificial materials under fluctuating conditions imposed by salivary flow, nutrient pulses, pH variation, oxygen gradients, host-derived substrates, and mechanical disturbance [10,11,12]. They predominantly persist as structured multispecies biofilms, in which co-aggregation, competition, signaling, and metabolic cross-feeding can generate microscale chemical heterogeneity [13,14]. However, it remains unclear whether microbiologically influenced corrosion (MIC) at oral material interfaces is primarily determined by particular microorganisms or instead reflects emergent functions of the biofilm community.

Orthodontic appliances introduce metallic surfaces into this ecosystem. Medical-grade 316L stainless steel (316L SS) is widely used in orthodontic devices and temporary anchorage components because of its mechanical performance, manufacturability, biocompatibility, and corrosion resistance [15,16,17]. Its corrosion resistance is conferred by a chromium-rich passive oxide film, but this film can be destabilized by acidic challenge, fluoride exposure, chloride-containing saliva, proteins, mechanical loading, and wear [18,19,20]. Biofilm colonization adds a further level of complexity: biofilms can concentrate extracellular polymeric substances, metabolites, ions, and corrosion products at the metal surface while establishing localized gradients in pH, oxygen, and redox potential [1,9,21]. Thus, corrosion of medical 316L SS in the oral environment cannot be inferred from bulk saliva chemistry or material properties alone.

MIC describes corrosion processes that are modified by microbial activity at material interfaces [2]. Established MIC mechanisms include local acidification, oxygen depletion, extracellular polymeric substance accumulation, mineral deposition, passive-film modification, metal-ion complexation, and changes in interfacial redox chemistry [22]. Although MIC has often been investigated through individual corrosive microorganisms or bulk microbial abundance, these researchers do not readily explain why distinct communities colonizing comparable surfaces can produce different corrosion outcomes [23,24,25]. In multispecies biofilms, one taxon may alter corrosion indirectly by supplying metabolites, removing inhibitory products, modifying local oxygen or pH conditions, or promoting the persistence and spatial organization of other members [1,3,6]. Functional complementarity and interspecies interactions may therefore influence corrosion even when no individual member is sufficient to generate the complete phenotype.

Flavins provide a tractable candidate link between microbial metabolism and interfacial redox processes. Riboflavin and related flavins can act as diffusible redox mediators in diverse microorganisms and, in some systems, facilitate extracellular electron transfer to extracellular substrates [26,27]. Riboflavin-associated processes have also been implicated in stainless steel MIC [25,28,29,30]. However, the ecological basis of extracellular riboflavin accumulation in multispecies oral biofilms remains unresolved. In particular, it is not known whether corrosion-active oral biofilms are characterized by a distinct community structure and metabolic complementarity, whether these properties are associated with extracellular riboflavin accumulation, or whether a simplified community can reproduce key features of a high-corrosion phenotype.

Here, we investigated oral microbiota-mediated corrosion of medical 316L SS as a community-level phenotype. We first compared subject-derived oral consortia incubated with medical 316L SS under standardized simulated oral conditions and evaluated metal release, electrochemical activity, and localized surface damage. We then compared planktonic and metal-associated biofilm communities using 16S rRNA gene profiling and functional prediction to identify community features associated with the attached state. Finally, we reconstructed a defined community to test whether community assembly was associated with enhanced biofilm formation, corrosion activity, and extracellular riboflavin accumulation. By integrating community profiling, defined-community reconstruction, electrochemical measurements, metabolite quantification, and surface characterization, this study examines how community assembly is associated with the emergence of corrosion-active oral biofilms and identifies extracellular riboflavin as a candidate redox-active mediator at the oral biofilm–metal interface. This study provides an ecological framework for understanding corrosion at oral biofilm–metal interfaces.

## 2. Materials and Methods

### 2.1. Study Design and Experimental Overview

This study examined whether variation in oral biofilm communities was associated with differential corrosion of medical 316L SS and whether a defined community could reproduce selected features of a high-corrosion phenotype. The study comprised four linked components. First, a retrieved orthodontic micro-implant of medical 316L SS was examined to document the co-occurrence of surface-associated biofilm and localized corrosion features. Second, oral microbial communities from 13 donors were incubated independently with medical 316L SS coupons under standardized simulated oral conditions, and corrosion was evaluated by dissolved metal release and electrochemical measurements. Third, planktonic and coupon-associated biofilm fractions were profiled by 16S rRNA gene amplicon sequencing to identify taxa and predicted functions associated with the metal-attached state. Finally, a defined community was assembled to test how community composition was associated with biofilm development, extracellular riboflavin accumulation, and corrosion-related endpoints. Riboflavin and roseoflavin were used as chemical perturbations to assess the association between extracellular riboflavin availability and corrosion activity.

### 2.2. Ethics Approval, Participant Recruitment, and Oral Sample Collection

The study protocol was approved by the Ethics Committee of the China Medical University Stomatological Hospital (Shenyang, China) (approval No. 2021-17, 26 October 2021). All participants provided written informed consent before enrolment.

Oral microbial samples were obtained from 13 adult participants (age range: 20–28 years, mean age: 23.5 ± 2.2 years; 7 males and 6 females; Table A1) recruited at the China Medical University Stomatological Hospital. Samples were collected from orthodontic micro-implant-associated plaque using sterile cotton swab and curette. To reduce major confounding effects on the oral microbiota, participants were excluded if they had received systemic antibiotics, probiotics, or antiseptic mouthwash within 14 days before sampling, or if they had predefined exclusion criteria, such as active periodontal disease, untreated caries, smoking, systemic disease, or pregnancy.

All samples were collected under standardized conditions and immediately stored at –80 °C until analysis. During transport, samples were maintained at 4 °C. Samples were handled individually throughout inoculum preparation and incubation; no donor samples were pooled for the subject-derived community experiments.

### 2.3. 316L SS Coupons and Pre-Treatment

Medical-grade 316L SS was used as the test material. Its nominal elemental composition (wt%) was 0.019 C, 0.43 Si, 1.18 Mn, 10.5 Ni, 16.78 Cr, 2.09 Mo, 0.032 P, 0.0006 S, and balance Fe. Coupons were machined to 10 mm × 10 mm × 5 mm. Before each experiment, coupon surfaces were sequentially ground with silicon-carbide abrasive papers from 240 to 1000 grit to standardize surface preparation. Coupons were then ultrasonically cleaned in deionized water and absolute ethanol for 15 min each, air-dried under sterile conditions, and finally sterilized by UV irradiation.

For electrochemical experiments, coupons were embedded in epoxy resin, leaving an exposed working area of 1 cm^2^. The exposed area was measured and used for current density normalization. Coupons were randomly assigned to experimental groups, and each coupon was used in only one independent culture–coupon experiment.

### 2.4. Culture Medium and Incubation Conditions

A modified artificial-saliva medium was used for subject-derived community incubations [9], defined-community experiments, and corrosion assays unless otherwise indicated. The medium contained (mg/L) NaCl, 125.6; KCl, 963.9; KH_2_PO_4_, 654.5; Na_2_SO_4_, 336.6; NH_4_Cl, 178; urea, 200; NaHCO_3_, 630.8; and CaCl_2_, 172. Yeast extract (4 g/L) and glucose (2 g/L) were added as sources of organic nutrients and growth factors. The pH was adjusted to 6.8 ± 0.1 with sterile phosphoric acid.

For anoxic incubations, the medium was sparged with high-purity nitrogen for 40 min before use and transferred into an anaerobic chamber to minimize oxygen exposure. Incubations were performed at 37 °C for 7 days under anaerobic conditions. The atmospheric condition was maintained using high-purity nitrogen.

### 2.5. Subject-Derived Oral Community Incubation and Corrosion-Phenotype Screening

For each donor, an individual oral-community inoculum was prepared using a standardized protocol involving suspension, vortexing, homogenization, dilution, or washing. Prior to inoculation, the inocula were standardized to an optical density at 600 nm (OD_600_) of 0.8 to ensure consistent starting biomass across all donor-derived cultures. The final inoculum volume was 1 mL per 10 mL culture volume.

Each incubation vessel contained one 316L SS coupon and 10 mL modified artificial-saliva medium. Inoculated vessels were incubated at 37 °C for 7 days under the conditions described above. Sterile medium containing a coupon served as the abiotic control in every batch. Each donor-derived community was tested in three independent culture–coupon systems per experiment, and the full experiment was repeated independently three times. In this design, the culture–coupon system was the experimental unit: the three systems within one experiment constituted technical replicates of the same donor-derived community, and the three repeated experiments, each initiated from the same frozen inoculum stock on separate occasions, constituted three independent biological replicates. For statistical analysis, the three technical replicates within each experiment were averaged first, and the reported values represent the mean ± SD of the three independent experiments (n = 3).

At the endpoint, culture supernatants were collected for quantification of dissolved Cr and Fe, and extracellular riboflavin. Planktonic and coupon-associated biofilm were recovered separately for microbial community analysis. Corrosion phenotype was assessed based on metal release, corrosion current density (*i*_corr_), and, where indicated, localized pit depth.

### 2.6. Recovery of Planktonic and Coupon-Associated Biofilm Fractions

At day 7, planktonic cultures were collected from each vessel prior to coupon removal. To harvest coupon-associated biomass, the coupons were gently rinsed three times with sterile PBS buffer to remove non-adherent cells. The remaining attached biofilm was recovered via vortexing and bead beating in PBS buffer for 5 min at 5 m/s. This identical recovery protocol was applied to all coupons. The recovered planktonic and biofilm fractions were either processed immediately or stored at −80 °C until DNA extraction. For each fraction, material from technical replicates was maintained separately.

### 2.7. DNA Extraction, 16S rRNA Gene Sequencing, and Sequence Processing

DNA was extracted from planktonic and coupon-associated biofilm samples using the DNeasy PowerSoil Kit (Qiagen, Hilden, Germany) according to the manufacturer’s instruction. The V3–V4 region of the bacterial 16S rRNA gene was amplified using primers 338F (5′–ACTCCTACGGGAGGCAGCAG–3′) and 806R (5′–GGACTACHVGGGTWTCTAAT–3′). PCR reactions were performed using ABI GeneAmp^®^ 9700 PCR thermocycler (ABI, Foster City, CA, USA). After PCR amplification and purification, the 16S rRNA gene was sequenced on an Illumina MiSeq PE300 platform (Illumina, San Diego, CA, USA). Raw reads were demultiplexed and quality-filtered using QIIME2 pipeline (v2023.7) [31]. Forward and reverse reads were merged, and chimeric sequences were removed. Taxonomic classification was performed using QIIME2 classifier against the Genome Taxonomy Database (GTDB) reference database (vr2.7.2) [32]. Taxonomic labels follow the GTDB naming convention, in which suffix codes (e.g., _A, _B, _D) distinguish genomically distinct lineages that share a genus or species name. All microbial community analyses were conducted in R (v4.0) using microeco (v2.3.0) [33].

### 2.8. Functional Inference and Genome-Informed Metabolic Analysis

Predicted functional potential of 16S rRNA gene profiles was inferred using PICRUSt2 (v2.6.3) with PICRUSt2-SC database [34]. Predicted pathway abundances were compared between planktonic and coupon-associated communities using Kruskal–Wallis.

For the community analysis, genome-scale metabolic models for OTUs were generated using CarveMe (v1.5.1) from GTDB reference database (vr2.7.2) [35]. Pairwise metabolic interaction potential was estimated using SMETANA (v1.1.0) under medium conditions designed to approximate the modified artificial-saliva medium [35]. Putative riboflavin biosynthesis and utilization functions were annotated using KEGG (https://www.genome.jp/kegg/, accessed on 1 June 2024) [36].

All PICRUSt2- and genome-model-derived results were interpreted as predictions of metabolic potential. They were used to generate hypotheses for experimental testing and were not interpreted as direct evidence of transcription, metabolite exchange, or extracellular electron transfer (EET).

### 2.9. Defined-Community Construction and Experimental Design

The defined community consisted of *Comamonas tsuruhatensis* (laboratory stock obtained from BNCC, Biejing, China, GCA_001571325.1), *Rhodococcus erythropolis* (laboratory stock obtained from BNCC, GCA_016598535.1), and *Thauera aromatica* (laboratory stock obtained from Mingzhou Bio, Ningbo, China, GCA_003030465.1). The three strains were selected because they were detected at higher relative abundance in coupon-associated communities and showed high predicted metabolic complementarity in the genome-informed analysis. The taxonomic identity of each strain was confirmed by 16S rRNA gene amplification and sequencing, and GTDB-based classification (GTDB-Tk (vr2.7.2) [32]) placed all three strains within the same GTDB genomic clusters as the corresponding taxa detected in the subject-derived communities.

Each strain was cultured separately in BHI medium at 37 °C under anaerobic conditions until reaching the mid-log phase (OD_600_ = 0.6). Cells were harvested by centrifugation at 5000× *g* for 5 min, washed 3 times with PBS buffer, and resuspended in modified artificial saliva. Cell suspensions were normalized to a density of 10^8^ CFU/mL.

Monocultures, all three pairwise combinations, and the three-member community were inoculated at an identical total starting cell density of 10^8^ cells/mL. In mixed communities, each constituent strain contributed equal cell numbers to the total inoculum. Each condition was incubated with 316L SS coupons for 7 days as described in Section 2.4 and Section 2.5. The subject-derived S3 community served as a reference community with a high corrosion-associated phenotype, because it showed the highest release of iron and chromium ions from 316L SS among the 13 donor-derived communities evaluated in the screening experiment.

### 2.10. Riboflavin and Roseoflavin Perturbation Experiments

Riboflavin and roseoflavin were obtained from Macklin (Shanghai, China) and prepared as stock solutions. Roseoflavin, a riboflavin analogue that can interfere with flavin-dependent metabolic processes [37], was used as a chemical perturbagen to examine changes associated with extracellular riboflavin availability; its potential broader effects on microbial physiology are considered in the Discussion (Section 4). Vehicle controls received the same final solvent concentration as compound-treated cultures. Riboflavin and roseoflavin were added at inoculation to final concentrations of 5 μg/mL and 5 mg/mL, respectively [25,26,28,38].

The following conditions were included where applicable: sterile medium, sterile medium supplemented with riboflavin, sterile medium supplemented with roseoflavin, untreated community, vehicle-treated community, community supplemented with riboflavin, and community treated with roseoflavin. Coupons were incubated for 7 days before electrochemical measurements and endpoint sampling.

### 2.11. Biomass, Viability, Growth, pH, and Community-Composition Controls

Biofilm structure and viability were assessed by confocal laser scanning microscopy (CLSM; LSM 900, Zeiss, Oberkochen, Germany) as described in Section 2.14. Total biofilm biomass was quantified using crystal violet staining, and surface-associated ATP levels were measured using an ATP fluorescence detector (UPF-10ATP, UP General, Beijing, China). Culture pH was measured. These measurements were used to evaluate whether differences in corrosion rate could be explained by gross changes in biomass, cell viability, or bulk pH.

### 2.12. Electrochemical Measurements

Electrochemical measurements were performed at 37 °C using a three-electrode configuration. The 316L SS coupon served as the working electrode, a saturated calomel electrode (SCE) as the reference electrode, and a platinum sheet as the counter electrode. Each glass cell contained 200 mL of experimental medium and was inoculated with 2 mL of microbial suspension at standardized inoculum. The exposed surface area of the working electrode was 1 cm^2^.

Open-circuit potential (OCP) and potentiodynamic polarization were measured using a Gamry Reference 600 potentiostat (Gamry Instruments, Warminster, PA, USA). OCP was monitored for 0.5 h before polarization measurements. Potentiodynamic polarization was recorded after 7 days of incubation from −0.3 to 1.2 V relative to OCP at 0.333 mV/s. Corrosion current density (*i*_corr_) was derived from Tafel extrapolation. The complete electrochemical measurement (0.5 h of OCP stabilization followed by the potentiodynamic scan) was finished within approximately 2 h, immediately after 7 days of biofilm maturation. Because this period is far shorter than the generation times of the dominant community members, meaningful changes in microbial community composition during the measurement were considered negligible, and community composition was characterized at the experimental endpoint (Section 2.7). Three independent experiments were performed for each condition; within each experiment, measurements from three technical replicate cells were averaged before pooling across experiments (n = 3).

### 2.13. Metal-Release Analysis and Extracellular Riboflavin Quantification

After incubation, culture supernatants were collected by centrifugation at 10,000× *g* for 5 min and filtration through 0.22 μm membranes. For metal analysis, samples were acidified using a HNO_3_–HClO_4_–HF mixture (2:1:2, *v*/*v*/*v*) and diluted as required. Dissolved Cr and Fe concentrations were quantified by inductively coupled plasma mass spectrometry (ICP–MS; Agilent 7850, Agilent Technologies, Santa Clara, CA, USA). Calibration curves were generated from certified standards, and blank medium values were subtracted from experimental measurements.

Extracellular riboflavin concentrations were determined by high-performance liquid chromatography (HPLC; UltiMate 3000, Thermo Scientific, Waltham, MA, USA) using a C18 column (150 × 4.6 mm, 5 μm; Bonna-Agela Technologies, Tianjin, China). Samples were protected from light during collection and analysis. The mobile phase comprised methanol and 0.05 M ammonium acetate buffer (pH 6.0) at 85:15 (*v*/*v*) and was delivered isocratically at 1.0 mL/min. Riboflavin was monitored at 445 nm and quantified against an external standard curve prepared with authentic riboflavin.

### 2.14. Biofilm Imaging and Surface Characterization

For CLSM, coupon-associated biofilms were stained with the LIVE/DEAD BacLight bacterial viability kit (Thermo Fisher Scientific, Waltham, MA, USA) according to the manufacturer’s instructions. Samples were imaged using a Zeiss LSM 900 confocal microscope (Zeiss, Oberkochen, Germany). Three-dimensional reconstructions were generated using ZEN software (https://www.zeiss.com/microscopy/us/products/software/zeiss-zen.html, accessed on 1 January 2025) (Zeiss), and biofilm thickness was calculated from z-stack images.

For corrosion-pit analysis, biofilms and corrosion products were removed following GB/T 4334.4-2000 [39]. Coupons were ultrasonically cleaned in ethanol and briefly exposed to a hydrofluoric-acid/nitric-acid solution as specified by the standard. The duration of chemical cleaning was standardized across samples to minimize alteration of the underlying substrate. Pit morphology and depth were then analyzed by CLSM.

### 2.15. Metal-Release Assay Without an Added Organic Carbon Source

To evaluate metal dissolution associated with the oral microbial community under conditions without added organic carbon, we performed a dedicated assay without external organic carbon sources or organic electron donors in the bulk medium. Briefly, anaerobic incubation media were prepared in a simulated-saliva–based inorganic formulation and supplemented with salts and trace components as in the main corrosion assays, while omitting all organic substrates (e.g., carbohydrates, organic acids, peptides, amino acids, or any other assimilable carbon sources). Under these conditions, the 316L SS coupon represented the only intentionally supplied solid-phase material with the potential to act as an electron donor in interfacial redox processes, while the input of exogenous organic electron donors was minimized.

For each condition, pre-cleaned 316L SS coupons were incubated at 37 °C under anoxic conditions with the following groups: (i) sterile control, (ii) S3 community, (iii) S3 + riboflavin, and (iv) S3 + roseoflavin. Riboflavin and roseoflavin were added at the concentrations used in the related supplementation experiments (final concentrations stated in the main Section 2.10). Incubations were conducted for the same duration as in the corresponding metal-release experiments. After incubation, culture liquids were collected, filtered (0.22 μm), and the dissolved Cr and Fe concentrations were quantified by ICP–MS. Solution pH was measured at the endpoint toassess whether supplementation substantially altered bulk acidity.

In this assay context, increased dissolved Cr/Fe release was interpreted as evidence of enhanced metal dissolution associated with microbial presence under oligotrophic conditions (i.e., in the absence of added organic carbon), rather than as direct evidence that 316L SS served as a biological electron donor in microbial metabolism. Changes observed following riboflavin supplementation and roseoflavin treatment were interpreted as supporting an association between extracellular riboflavin availability and metal corrosion activity. All measurements were performed in three independent experiments (n = 3), each comprising triplicate technical systems that were averaged before pooling; results are reported as mean ± SD.

### 2.16. Statistical Analysis

Data are presented as the mean ± SD of three independent experiments (n = 3) unless otherwise stated. Normality was assessed using the Shapiro–Wilk test, and homogeneity of variances was evaluated using Levene’s test. For two-group comparisons, Student’s *t*-test (normally distributed data) or the Mann–Whitney U test (non-normally distributed data) was used. For multiple-group comparisons, one-way analysis of variance (ANOVA) followed by Tukey’s HSD post hoc test was used for normally distributed data, whereas the Kruskal–Wallis test followed by Dunn’s post hoc test was used for non-normally distributed data. Differences in microbial community composition between planktonic and biofilm-associated fractions were evaluated by permutational multivariate analysis of variance (PERMANOVA; adonis2 with 999 permutations) based on Bray–curtis dissimilarity. Differentially enriched taxa were identified using LEfSe (v4.6.0) (LDA score > 2, *p* < 0.05). Correlations between extracellular riboflavin concentrations and dissolved Cr or Fe were assessed using Spearman’s rank correlation. Statistical significance was defined as *p* < 0.05. In all figures, the number of observations and the statistical tests applied are stated in the corresponding figure legends. All statistical analyses were performed in R (v4.0) using the microeco (v2.3.0) [33] and vegan (v2.7.5) packages.

## 3. Results

### 3.1. Subject-Derived Oral Communities Are Associated with 316L SS Corrosion

**Figure 1:**
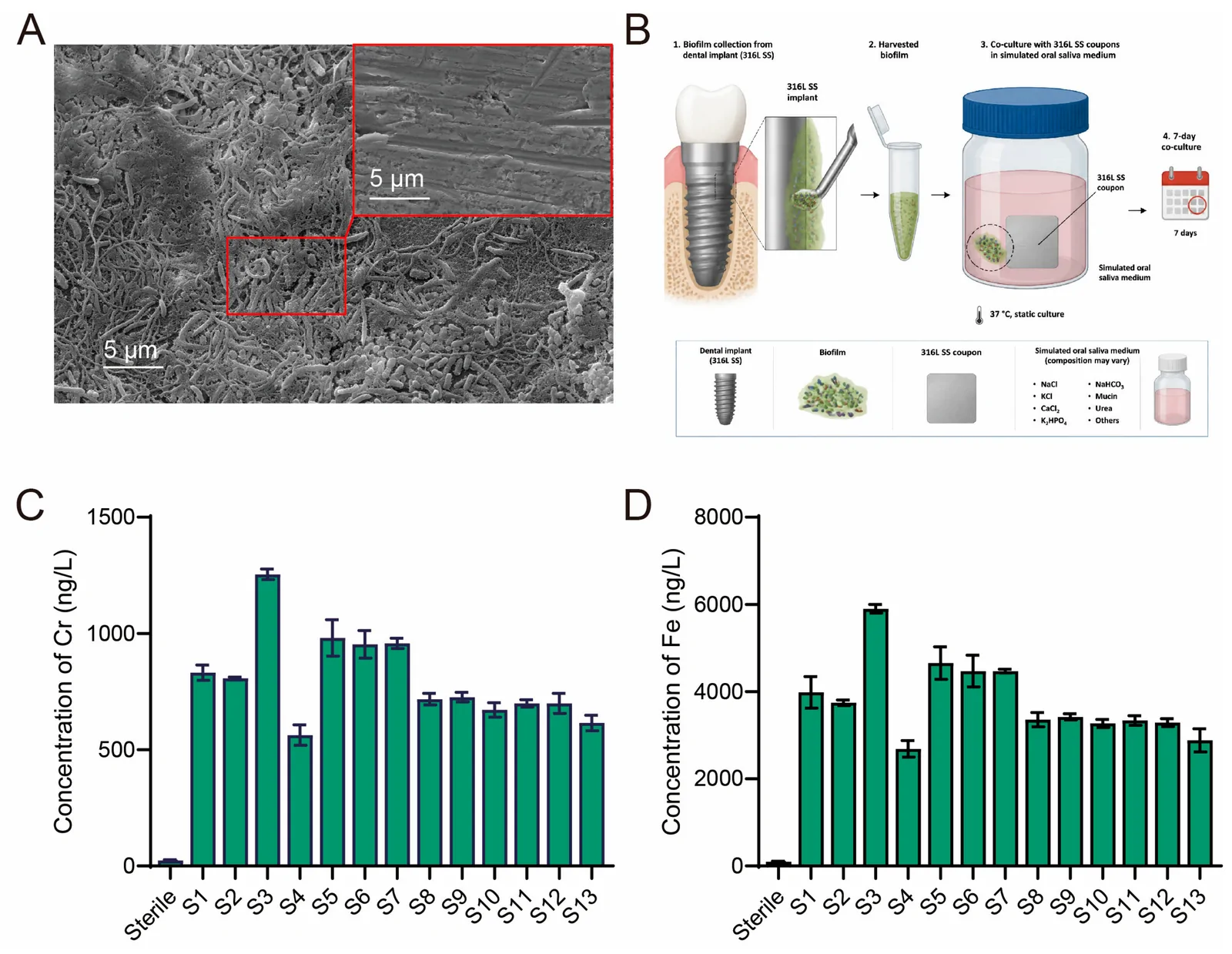
Clinical observation and ex vivo assessment of 316L SS corrosion associated with subject-derived oral biofilm communities. (**A**) Representative SEM image of a multispecies biofilm on a retrieved orthodontic micro-implant. The inset (red box) shows the underlying 316L SS surface after removal of biomass and corrosion products, revealing localized corrosion features. (**B**) Experimental workflow. Biofilms recovered from dental implants were used to inoculate simulated oral saliva medium containing 316L SS coupons. Cultures were maintained at 37 °C for 7 days before analysis. (**C**,**D**) ICP–MS quantification of dissolved (**C**) Cr and (**D**) Fe from 316L SS in sterile controls and cultures inoculated with oral biofilm communities from 13 subjects (S1–S13). For each condition, data are presented as the mean ± SD of three independent experiments (n = 3). Within each independent experiment, three technical replicates were averaged to obtain a single value.

To examine whether biofilm formation on retrieved orthodontic micro-implant anchorage devices was associated with implant corrosion, the surface of a representative retrieved implant was examined by SEM. A dense multispecies biofilm embedded in an extracellular polymeric substance matrix was observed on the implant surface (Figure 1A). Following removal of biomass and corrosion products, localized surface corrosion features were visible on the underlying metal. These clinical observations suggested an association between oral biofilms and corrosion of medical-grade 316L SS implants.

To further examine this clinically suggested association, we next evaluated 316L SS corrosion in the presence of subject-derived oral microbial communities. Oral consortia derived from 13 independent subjects (S1–S13) were incubated with 316L SS coupons in simulated saliva for 7 days (Figure 1B). Dissolved Cr and Fe in the bulk medium were quantified by ICP–MS as indicators of metal corrosion and dissolution (Figure 1C,D). Sterile controls showed only trace concentrations of Cr and Fe, whereas every inoculated community yielded markedly greater concentrations of both metals (Figure 1C,D). Cr concentrations varied across the subject-derived communities, ranging from approximately 550 ng/L in S4 to 1250 ng/L in S3 (Figure 1C). Fe concentrations showed a similar pattern, ranging from approximately 2700 ng/L in S4 to 5900 ng/L in S3 culture (Figure 1D). S3 consortium exhibited the highest concentrations of both Cr and Fe, whereas S4 showed the lowest metal-release phenotype among the inoculated communities. Thus, oral biofilm communities increased corrosion of 316L SS relative to the sterile condition, while the magnitude of this effect varied among subject-derived communities.

### 3.2. 316L SS-Associated Biofilms Exhibited Trends Toward Compositional Differences from Planktonic Communities

To characterize microbial characteristics during the 316L SS interface corrosion process, 16S rRNA gene sequencing was performed on paired planktonic and surface-associated biofilm fractions recovered from the 13 subject-derived incubation systems.

**Figure 2:**
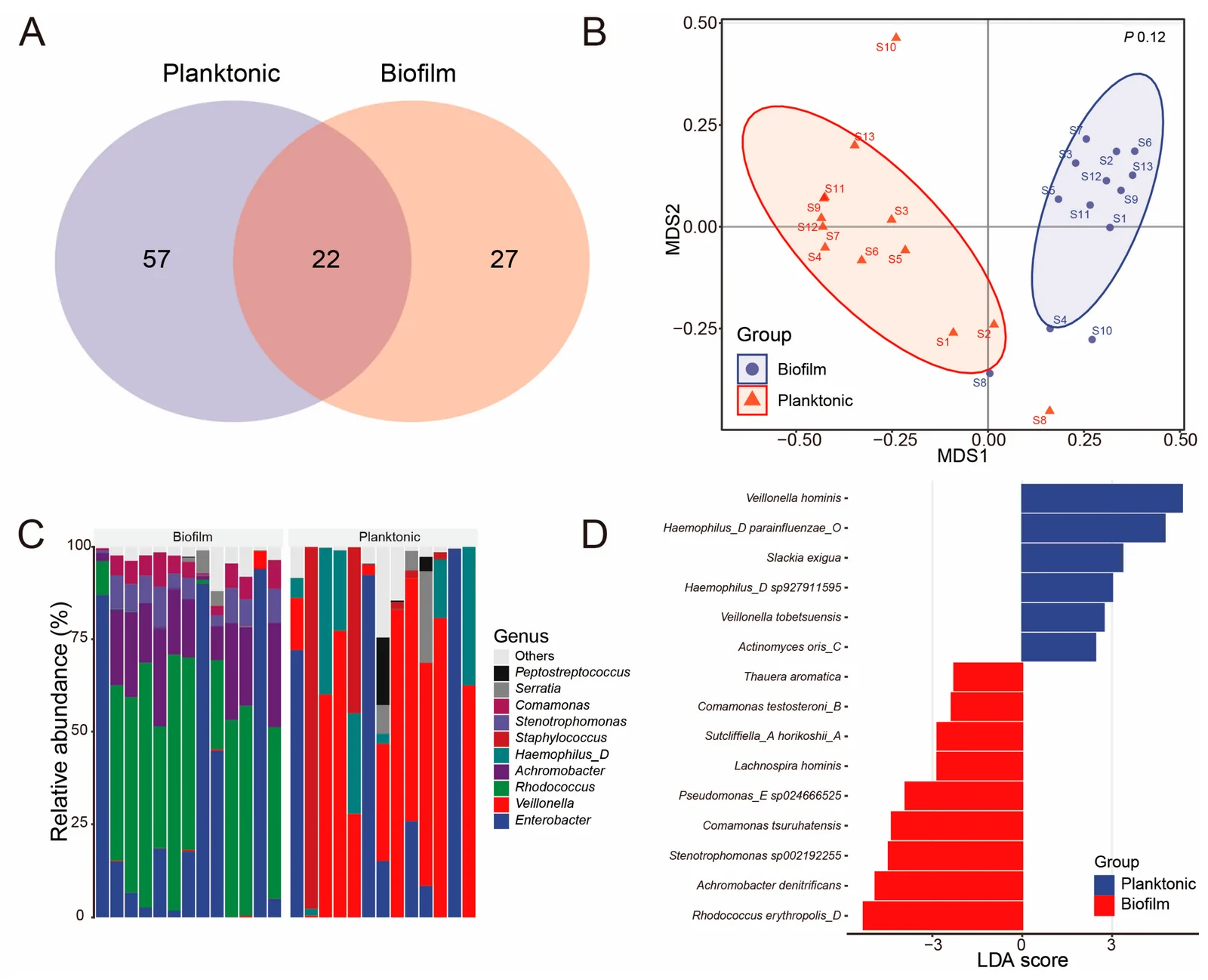
Taxonomic features of planktonic and 316L SS-associated biofilm communities in subject-derived incubation systems. (**A**) Venn diagram showing shared and fraction-specific OTUs in planktonic and biofilm fractions (**B**) NMDS ordination based on Bray–curtis dissimilarity. Each point represents a community derived from one subject. (**C**) Genus-level relative-abundance profiles of individual planktonic and biofilm-associated communities. (**D**) Taxa differentially enriched between the planktonic and biofilm fractions, identified by LEfSe analysis (*p* < 0.05, LDA score > 2). Blue bars indicate enrichment in planktonic communities, and red bars indicate enrichment in 316L SS-associated biofilms. PERMANOVA (*p* = 0.12) and LEfSe (LDA score > 2, *p* < 0.05) were applied as indicated.

Operational taxonomic unit (OTU) membership differed between the two fractions. The planktonic and biofilm fractions contained 57 and 27 unique OTUs, respectively, whereas 22 OTUs were shared between fractions (Figure 2A). NMDS based on Bray–curtis dissimilarity showed a trend towards separation between planktonic and biofilm-associated communities. However, this difference was not statistically supported by PERMANOVA (PERMANOVA, *p* = 0.12; Figure 2B). Biofilm samples nevertheless showed a comparatively tighter distribution in the ordination space, suggesting that communities developing on 316L SS showed a trend toward reduced dispersion across subject-derived cultures compared with their corresponding planktonic communities.

Genus-level profiles further indicated differences in the taxa represented in the two fractions (Figure 2C). Planktonic communities were dominated primarily by *Veillonella* and *Haemophilus*_D in several subject-derived cultures, whereas surface-associated biofilms showed greater relative abundances of *Enterobacter*, *Rhodococcus*, and *Comamonas*. Consistent with these compositional patterns, LEfSe analysis identified *Veillonella hominis*, *Haemophilus*_D *parainfluenzae*, *Slackia exigua*, *Haemophilus*_D sp. 329791195, *Veillonella tobetsuensis*, and *Actinomyces oris* as taxa enriched in the planktonic fraction. In contrast, *Rhodococcus erythropolis*_D, *Achromobacter denitrificans*, *Stenotrophomonas* sp. 90219255, *Comamonas tsuruhatensis*, *Pseudomonas* sp. 024666525, *Lachnospira hominis*, *Sutcliffiella halophila*, *Comamonas testosteroni*_B, and *Thauera aromatica* were enriched in the 316L SS-associated biofilm fraction (Figure 2D; *p* < 0.05). The enrichment of these taxa in 316L SS-associated biofilms suggests that biofilm development at the metal interface may be accompanied by taxon-level differences potentially relevant to the observed metal-dissolution phenotype.

Across the 13 subject-derived systems, *Desulfobulbus oralis* was the only taxon potentially associated with sulfate reduction detected. It was found only in the planktonic community of subject S4 (1/13, 7.7%) at 0.12% relative abundance, and was absent from the remaining planktonic communities and all 13 coupon-associated biofilm communities (Table A2). Thus, 12 of the 13 subjects (92.3%) showed no detectable *D. oralis* in the planktonic fraction, and all 13 subjects (100%) showed no detectable *D. oralis* in the coupon-associated biofilm fraction (Table A2). Spearman analyses yielded *r* = −0.46 for Cr release and *r* = −0.46 for Fe release (Table A2); however, these coefficients were not interpreted because detection in only one subject provided insufficient variation for robust analysis. Overall, taxa potentially associated with sulfate reduction were detected only sporadically and at very low relative abundance (Table A2).

### 3.3. Biofilm-Associated Functional Potential and Extracellular Riboflavin Availability Were Associated with Enhanced 316L SS Corrosion Activity

**Figure 3:**
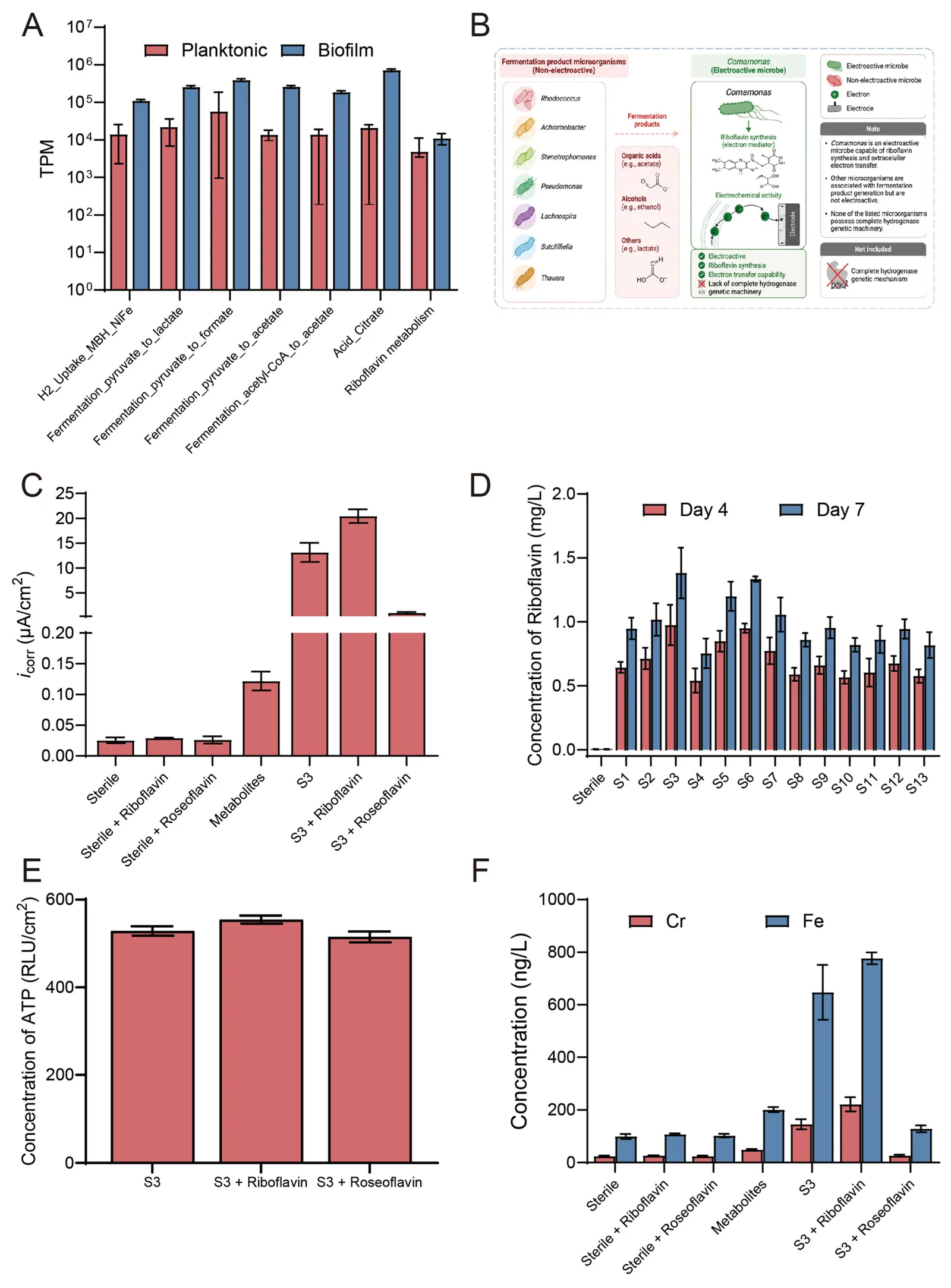
Predicted functional potential, extracellular riboflavin accumulation, and corrosion-associated activity in subject-derived oral biofilm communities. (**A**) Predicted abundances of selected functional pathways in planktonic and 316L SS-associated biofilm communities, determined using PICRUSt2. (**B**) Schematic summary of predicted taxon–function associations among taxa enriched in the biofilm fraction. Taxa associated with predicted fermentation-related functions are shown on the left, whereas *Comamonas* is shown as a taxon with predicted riboflavin-biosynthesis capacity. (**C**) *i*_corr_ of 316L SS coupons in sterile controls, sterile controls supplemented with riboflavin or roseoflavin, cell-free S3 culture metabolites, and the S3 community with or without riboflavin or roseoflavin supplementation. (**D**) Extracellular riboflavin concentrations in sterile controls and cultures inoculated with communities from subjects S1–S13 after 4 and 7 days of incubation. (**E**) Surface-associated ATP concentrations measured on 316L SS coupons after incubation with S3, S3 supplemented with riboflavin, or S3 treated with roseoflavin. (**F**) Dissolved Cr and Fe concentrations in the indicated experimental groups, quantified by ICP–MS. In this assay, no organic carbon source was added, and metal release was used as an indicator of microbially associated metal dissolution under oligotrophic conditions. This design does not by itself demonstrate that the steel served as a biological electron donor. Data are presented as mean ± SD from three independent experiments (n = 3).

To identify predicted functional features associated with 316L SS interfacial corrosion in the presence of oral microbial communities, predicted metabolic profiles were compared between planktonic and biofilm-associated communities using PICRUSt2. Relative to planktonic communities, biofilm-associated communities showed higher predicted abundances of functions related to membrane-bound [NiFe]-hydrogenase, pyruvate metabolism, acetyl-CoA-to-acetate conversion, citrate metabolism, and riboflavin metabolism (Figure 3A). Taxon–function mapping further indicated that biofilm-enriched taxa, including *Rhodococcus*, *Achromobacter*, *Stenotrophomonas*, *Pseudomonas*, *Lachnospira*, *Sutcliffiella*, and *Thauera*, were associated with predicted fermentation-related functions. In contrast, *Comamonas* was associated with predicted riboflavin-biosynthesis capacity (Figure 3B). Collectively, these results suggest that biofilm-associated communities harbor predicted functional capacities related to fermentation and riboflavin metabolism, which may be associated with increased 316L SS corrosion rates.

To test whether soluble microbial products contributed to the corrosion process, the S3 consortium, which showed the highest Cr and Fe release among the 13 subject-derived consortia (Figure 1B–D), was selected for further evaluation. The selection was based on the primary screening endpoint of dissolved Cr/Fe release, which directly quantifies metal corrosion and dissolution; the subsequent multi-parameter characterization (electrochemistry, pit morphology, biofilm thickness, surface ATP, pH, extracellular riboflavin, and the no-added-organic-carbon assay) was performed for this representative high-release consortium. Cell-free metabolites collected from the S3 consortium increased the corrosion current density (*i*_corr_) from 0.024 µA/cm^2^ in the sterile control to 0.12 µA/cm^2^ (Figure 3C). However, this value was substantially lower than that measured for the intact S3 consortium (13.1 µA/cm^2^). These results demonstrate that, while soluble culture-derived metabolites could increase the corrosion rate of 316L SS, they alone cannot account for the severe corrosion induced by the complete consortium.

**Figure A1:**
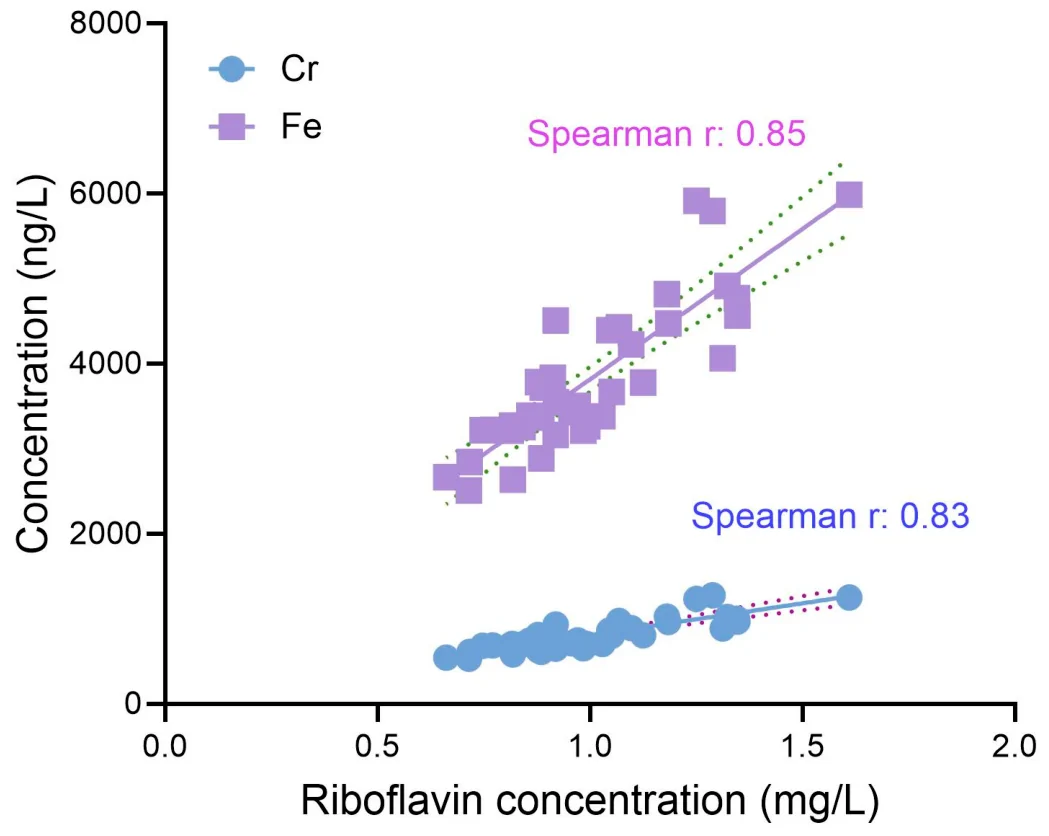
Spearman correlation between extracellular riboflavin and 316L SS dissolution in subject-derived consortia. Scatter plots showing the relationship between extracellular riboflavin concentration (mg/L) and dissolved concentrations of Cr (blue circles, Spearman *r* = 0.83) and Fe (purple squares, Spearman *r* = 0.85) across the 13 subject-derived communities. Each point represents one subject-derived consortium. Dotted lines indicate the correlation trend.

Given the predicted prominence of riboflavin metabolism at the biofilm–steel interface and the corrosion-associated effects observed with cell-free culture-derived products, extracellular riboflavin was quantified in supernatants from all subject-derived communities. While remaining undetectable in the sterile controls, riboflavin was detected across all inoculated systems (Figure 3D). Riboflavin concentrations varied among communities and were generally higher after 7 days than after 4 days of incubation, indicating its accumulation over the incubation period. Notably, the S3 consortium showed among the highest extracellular riboflavin concentrations at day 7. To evaluate the association between extracellular riboflavin availability and corrosion-associated metal dissolution across the subject-derived communities (Figure 1B–D), we performed correlation analyses between extracellular riboflavin and dissolved Cr and Fe. Across all consortia, the extracellular riboflavin concentration was positively correlated with both dissolved Cr and Fe concentrations (Spearman *r* = 0.83, *p* < 0.05 for Cr; *r* = 0.85, *p* < 0.05 for Fe; Figure A1). These findings indicate that higher extracellular riboflavin availability is associated with increased 316L SS metal corrosion, suggesting a possible contribution of riboflavin-associated interfacial electron transfer to oral microbiota-associated corrosion.

To evaluate whether riboflavin availability was associated with 316L SS corrosion, sterile controls and the S3 consortium were supplemented with riboflavin or roseoflavin. Surface-associated ATP concentrations were broadly similar among the untreated S3, S3 + riboflavin, and S3 + roseoflavin groups (Figure 3E). Overall, riboflavin or roseoflavin addition did not markedly alter surface-associated ATP levels. In sterile systems, riboflavin and roseoflavin induced little change in *i*_corr_ (Figure 3C). In contrast, riboflavin supplementation increased the *i*_corr_ of the S3 consortium from 13 to 20 μA/cm^2^, whereas roseoflavin treatment reduced *i*_corr_ to 0.2 μA/cm^2^. These results suggest that extracellular riboflavin availability is associated with altered corrosion activity of 316L SS in the S3 system.

**Figure A2:**
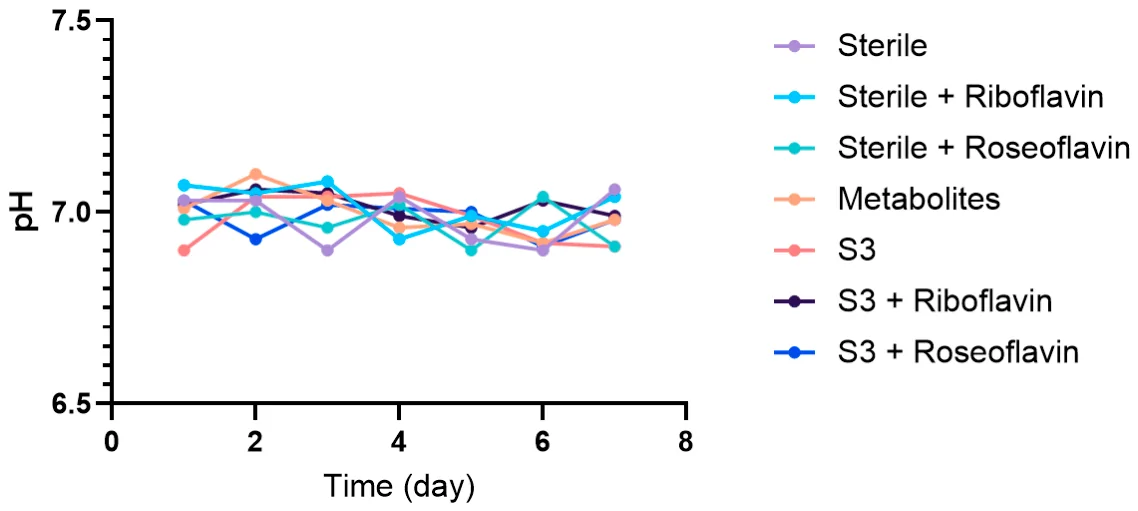
pH dynamics during corrosion assays under riboflavin and roseoflavin perturbations. Time-course measurement of solution pH from day 1 to day 7 across experimental groups: Sterile, Sterile + Riboflavin, Sterile + Roseoflavin, Metabolites (cell-free metabolites from S3), S3 (subject-derived consortium), S3 + Riboflavin, and S3 + Roseoflavin. Data are shown as mean values across time points.

To further assess the relationship between riboflavin availability and interfacial corrosion under oligotrophic conditions, Cr and Fe release was quantified using the assay described in Section 2.15, in which no organic carbon source was added and 316L SS represented the only intentionally supplied solid-phase material with the potential to act as an electron donor in interfacial redox processes. Relative to sterile controls, the S3 consortium significantly accelerated the release of both Cr and Fe (Figure 3F), consistent with enhanced metal dissolution associated with microbial activity in the absence of added organic carbon. Riboflavin supplementation further increased the concentrations of both dissolved metals, whereas roseoflavin treatment decreased Cr and Fe release relative to the untreated S3 community. Across all experimental groups, pH values remained near neutral and did not differ appreciably from controls (Figure A2). These findings support the view that oral microbiota-associated corrosion at the 316L SS interface is associated with extracellular riboflavin availability, consistent with a proposed riboflavin-linked interfacial redox process.

Collectively, the predicted functional profiles, extracellular riboflavin measurements, electrochemical responses, and metal-release data indicate that biofilm-associated communities are linked to fermentation- and riboflavin-related functional potential associated with enhanced 316L SS corrosion. In the S3 system, extracellular riboflavin availability was associated with elevated *i*_corr_ and accelerated 316L SS metal dissolution. These findings support an association between extracellular riboflavin availability and oral microbiota-associated corrosion at the 316L SS interface.

### 3.4. Riboflavin-Linked Interspecies Interactions Are Associated with Enhanced Oral Microbiota-Mediated Corrosion of 316L SS

**Figure 4:**
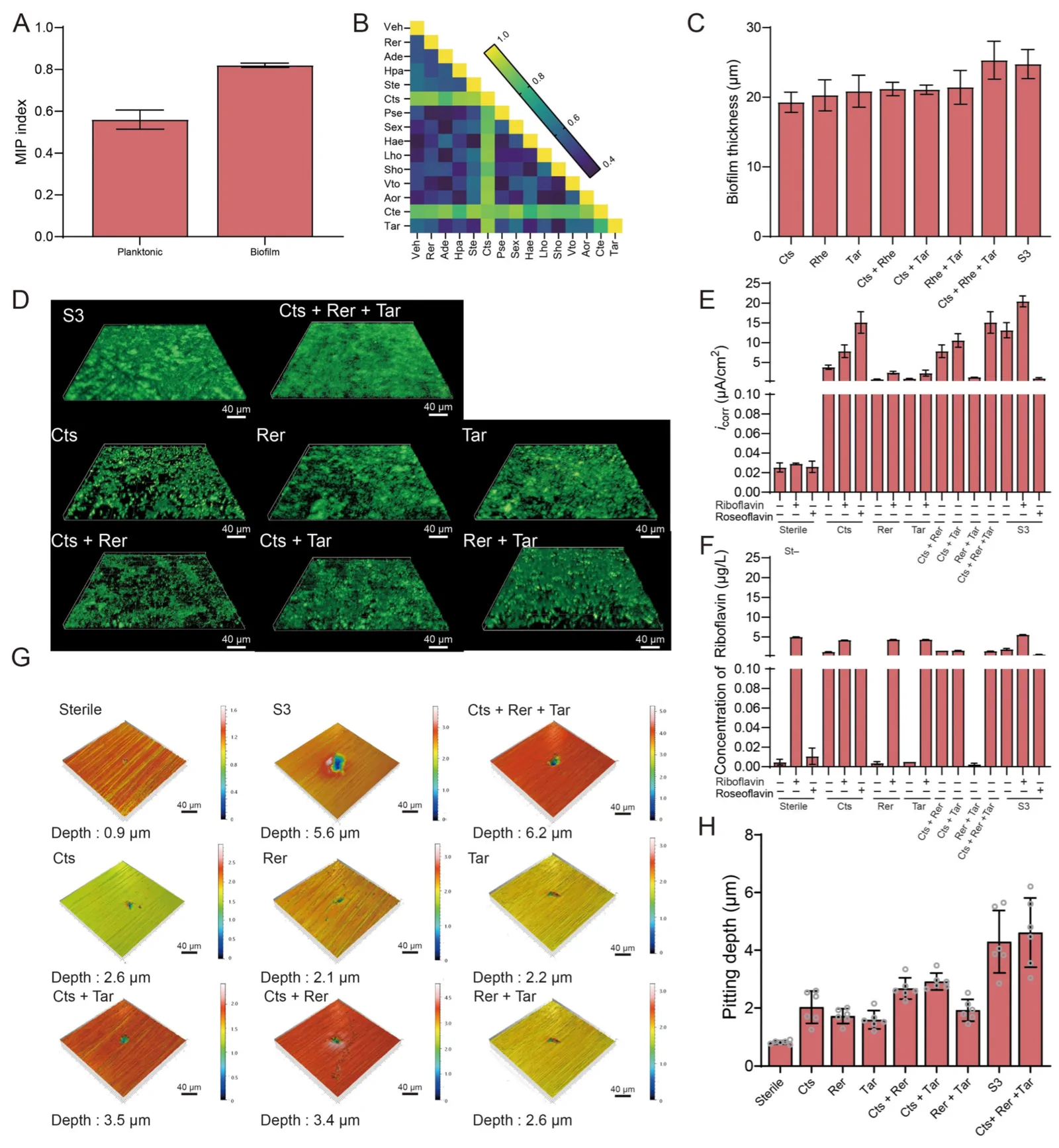
Genome-based metabolic interaction potential and biofilm–corrosion phenotypes of a defined three-strain consortium associated with riboflavin availability. (**A**) Mean metabolic interaction potential (MIP) of planktonic and biofilm communities, calculated from genome-based pairwise cross-feeding predictions. (**B**) Pairwise MIP matrix for taxa detected in the biofilm-associated community; higher values indicate greater predicted metabolic complementarity. (**C**) Biofilm thickness measured on day 7. (**D**) Representative three-dimensional CLSM reconstructions of biofilms formed on 316L SS coupons by the subject-derived S3 consortium, monocultures of *C. tsuruhatensis* (Cts), *R. erythropolis* (Rer), and *T. aromatica* (Tar), dual-species cultures, and the Cts + Rer + Tar consortium after 7 days of incubation. (**E**) *i*_corr_ of monocultures, dual-species cultures, the Cts + Rer + Tar consortium, and S3 under basal conditions and after riboflavin or roseoflavin supplementation. (**F**) Extracellular riboflavin concentrations measured by HPLC in the corresponding cultures. (**G**) Representative three-dimensional profilometry maps following removal of biomass and corrosion products. (**H**) Statistical analysis of pit depths for the indicated cultures. Data are presented as mean ± SD (n = 3). Statistical tests used are described in Section 2.16.

**Figure A3:**
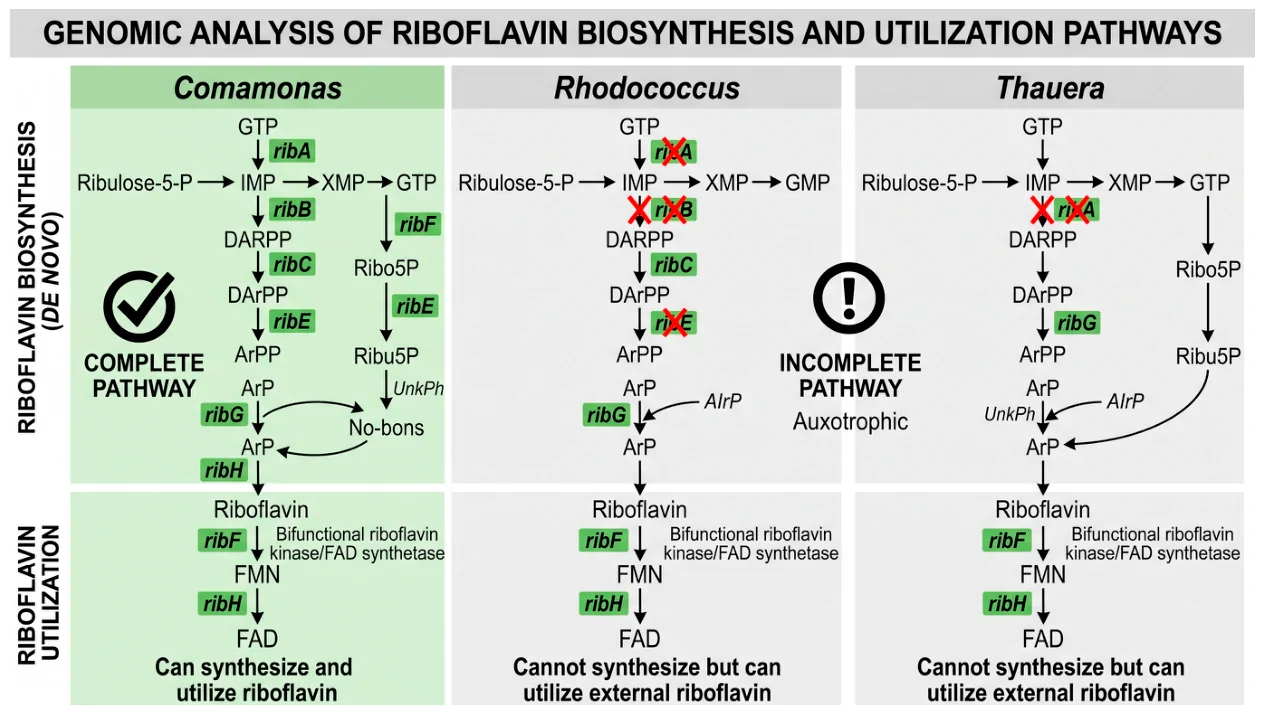
Genome-informed riboflavin biosynthesis and utilization potential in the reconstructed consortium strains. Schematic pathways for riboflavin synthesis and usage predicted from genome annotations in *Comamonas*, *Rhodococcus*, and *Thauera*. Green annotations indicate components of a complete riboflavin biosynthesis pathway (**left**) or missing/incomplete steps ((**middle**) and (**right**)). Red X marks denote pathway gaps, and the lower panels summarize whether each strain is predicted to synthesize riboflavin and/or utilize external riboflavin. The overall scheme supports predicted riboflavin exchange potential within the multi-strain community.

Metabolic interactions may shape biofilm development at the metal interface and thereby influence corrosion [1,5,6], we therefore evaluated metabolic interaction potential (MIP) using genome-based pairwise cross-feeding predictions. Biofilm–associated communities showed a higher mean MIP than planktonic communities (0.82 ± 0.01 vs. 0.56 ± 0.05, respectively; Figure 4A), indicating greater predicted metabolic complementarity among taxa within the biofilm. Pairwise MIP analysis of biofilm-associated taxa further identified predicted metabolic interactions between *Comamonas* and several biofilm-enriched taxa, including *Rhodococcus* and *Thauera* (Figure 4B). Genome annotation indicated that *Comamonas* harbored genes consistent with predicted riboflavin-biosynthesis capacity, whereas *Rhodococcus* and *Thauera* lacked a complete predicted riboflavin-biosynthetic pathway but retained genes potentially associated with riboflavin utilization (Figure A3). Together with the taxon–function associations in Figure 3B, these computational predictions suggest potential metabolic complementarity involving riboflavin-related functions among biofilm-associated taxa.

To experimentally examine whether the predicted riboflavin-linked metabolic relationships were associated with biofilm formation and corrosion, we constructed a defined three-strain consortium as a simplified model of the oral microbiota: *C. tsuruhatensis* (Cts), *R. erythropolis* (Rer), and *T. aromatica* (Tar). These strains were selected based on their enrichment in the biofilm fraction and their high predicted pairwise metabolic complementarity. The subject-derived S3 consortium, which showed the highest Cr and Fe release among the 13 subject-derived consortia, was used as a comparator. Consistent with a cooperative community structure, LIVE/DEAD-stained CLSM analyses showed that after 7 days, Cts, Rer, and Tar monocultures formed sparse, discontinuous surface aggregates, while dual-species combinations displayed only moderate surface coverage and limited three-dimensional maturation (Figure 4C,D). In contrast, the Cts + Rer + Tar consortium formed a dense and continuous biofilm architecture closely resembling that of S3 community, and biofilm thickness measurements supported the dominance of the triple consortium over monocultures and most dual-species cultures (Figure 4C).

We next assessed whether the defined consortium could recapitulate the corrosion phenotype associated with riboflavin availability observed in the S3 community. Among the monocultures, Cts showed the highest *i*_corr_ of 3.8 μA/cm^2^, whereas Rer and Tar displayed substantially lower values. The dual-species consortia displayed intermediate corrosion activities, with Cts-containing combinations generally showing higher *i*_corr_ values than Cts alone or the Rer + Tar consortium. Under basal conditions, the Cts + Rer + Tar consortium resulted in a markedly elevated *i*_corr_ and approached the *i*_corr_ observed for S3 (Figure 4E). Importantly, riboflavin and roseoflavin supplementation were associated with opposite corrosion responses: riboflavin supplementation increased *i*_corr_ value, whereas roseoflavin decreased *i*_corr_ value relative to the corresponding baseline cultures. The most pronounced corrosion responses were observed in Cts-containing consortia, which were associated with higher extracellular riboflavin availability, as well as in the S3 community (Figure 4E).

Consistent with these electrochemical trends, extracellular riboflavin quantification indicated that riboflavin accumulation was largely observed in Cts-containing cultures. Riboflavin was detected in Cts-containing systems and reached the highest levels in Cts + Rer + Tar, as well as in S3, whereas riboflavin levels in Rer, Tar, and the Rer + Tar combination were minimal (Figure 4F). Furthermore, roseoflavin supplementation reduced extracellular riboflavin concentrations in Cts-containing systems.

**Figure 5:**
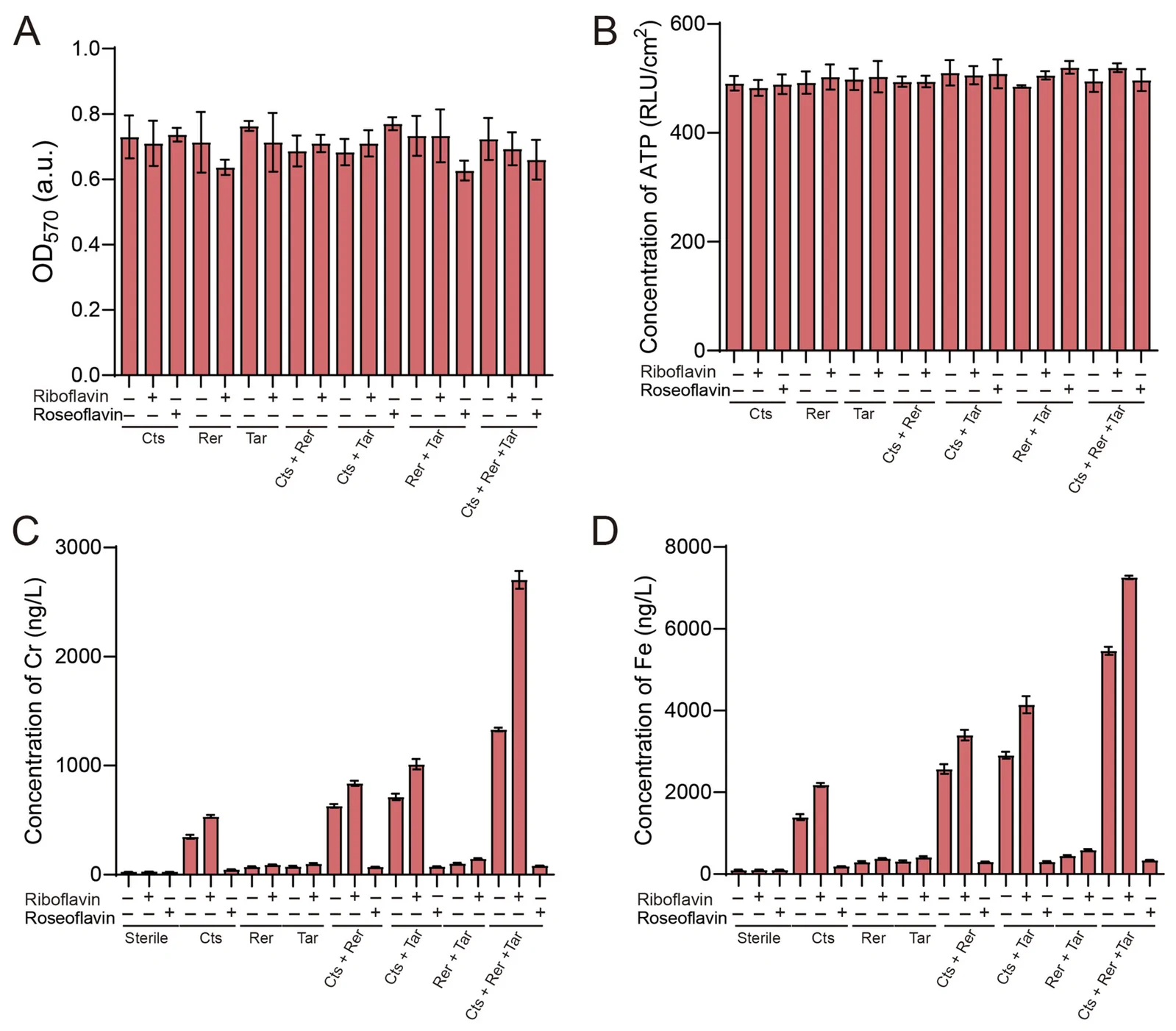
Riboflavin and roseoflavin effects on biofilm biomass, surface-associated ATP levels, and dissolved metal release in the defined community (**A**) Crystal violet staining as a proxy for total biofilm biomass (measured as OD_570_) across monocultures, pairwise consortia, the three-strain consortium, and the sterile control, with or without supplementation of riboflavin or roseoflavin. (**B**) Surface-associated ATP concentration corresponding to the same experimental groups and supplementation conditions. (**C**) Dissolved Cr concentration and (**D**) dissolved Fe concentration measured in the corrosion medium after incubation under the indicated conditions. Riboflavin and roseoflavin were added as indicated (±) for each group. Data are presented as mean ± SD (n = 3). Statistical tests used are described in Section 2.16.

Corrosion morphology and dissolution outcomes further supported an association between riboflavin availability and corrosion-related phenotypes. CLSM showed relatively uniform surface topography in the sterile control, whereas Cts-containing cultures induced progressively deeper and more extensive pit damage. Among the defined communities, the triple consortium induced the deepest pits, matching the pitting severity observed for S3 (Figure 4G,H). At the level of dissolved metals, re-tested assays confirmed that riboflavin supplementation increased Cr and Fe release, whereas roseoflavin suppressed metal dissolution in parallel with the electrochemical readouts (Figure 5C,D). Notably, these corrosion changes were not accompanied by a generalized increase in biofilm biomass or overall surface metabolic activity: riboflavin and roseoflavin produced little effect on surface-associated biomass and ATP levels across the corresponding groups (Figure 5A,B). Together, these results suggest that riboflavin-associated corrosion responses vary with species composition and are not explained solely by large changes in biofilm thickness.

Collectively, these reconstruction experiments suggest that the presence of Cts is associated with greater extracellular riboflavin availability, and that its presence in combination with Rer and Tar is associated with increased riboflavin accumulation, enhanced biofilm maturation, and greater corrosion-associated activity. In line with the subject-derived datasets, riboflavin availability was associated with metal dissolution, supporting a proposed riboflavin-linked model involving species-dependent metabolic interactions that may contribute to 316L SS corrosion at the oral microbiota–metal interface.

## 4. Discussion

Orthodontic micro-implants fabricated from 316L SS are exposed to a chemically and microbiologically heterogeneous oral environment. Although corrosion of stainless steel orthodontic devices has been documented in clinical retrieval and in vitro studies [9,23,24], the extent to which oral microbial community composition and community-level metabolism shape corrosion outcomes remains incompletely understood. Here, we extend the MIC framework by demonstrating that subject-derived oral consortia markedly accelerate 316L SS corrosion under controlled ex vivo conditions. The pronounced variation in corrosion-associated outcomes among subject-derived communities further suggests that microbial composition and functional potential may contribute to inter-individual differences in corrosion behavior. Collectively, these findings motivate a community-level framework for predicting microbiota-associated corrosion resistance at oral biomaterial interfaces.

Previous work has mainly attributed intra-oral corrosion to physicochemical factors, including acidic pH, fluoride exposure, chloride-containing saliva, oxygen gradients, and mechanical disruption of the passive film [40,41]. MIC has more recently been recognized as a potential contributor to implant corrosion and degradation in the oral cavity [9,23,24]. Subgingival microbiota have been reported to colonize 316L SS and promote localized pitting through acidic metabolites and electron-transfer-associated processes [9,23,24]. Consistent with these concepts, our analyses indicate that corrosion-associated communities possess predicted functional capacities enriched for fermentation-related metabolism (e.g., pyruvate metabolism, acetyl-CoA-to-acetate conversion, citrate metabolism) and riboflavin metabolism. Importantly, culture-derived, cell-free metabolites from the high-corrosion S3 consortium increased the *i*_corr_, but the effect was substantially weaker than that produced by the intact S3 consortium. This difference supports the view that fermentation products alone are unlikely to fully account for the high-corrosion phenotype and that community-level organization, such as biofilm development, redox gradients, extracellular polymeric substances, and interfacial redox processes that may involve electron transfer, likely contributes to the corrosion process.

Beyond differences in microbial composition, our results suggest that the 316L SS surface may exert niche-filtering effects during interface corrosion, although the overall separation between planktonic and biofilm-associated communities was not statistically significant (PERMANOVA, *p* = 0.12). Although planktonic and attached-biofilm fractions contained 57 and 27 unique OTUs, respectively, with only 22 OTUs shared between fractions, ordination based on Bray–Curtis dissimilarity showed a trend toward separation. Notably, biofilm samples displayed tighter clustering in ordination space than planktonic communities, consistent with reduced variability of communities surface-associated with the metal interface relative to the heterogeneous donor-associated baseline. Genus-level profiles further supported fraction-specific enrichment: planktonic communities were dominated primarily by *Veillonella* and *Haemophilus*_D, whereas 316L SS-associated biofilms showed greater relative abundances of *Enterobacter*, *Rhodococcus*, and *Comamonas*, with significant enrichment of taxa including *R. erythropolis*_D, *A. denitrificans*, *C. tsuruhatensis*, and *T. aromatica*. Together, the tighter clustering and consistent enrichment patterns are suggestive of selective enrichment of functional taxa at the metal interface, although these patterns should be interpreted cautiously given the lack of statistical support for community-level separation (PERMANOVA, *p* = 0.12).

SRBs are widely recognized as potential contributors to MIC [42,43,44,45]. However, the abundance of SRB does not necessarily correlate with the measured corrosion rate. Their effects may depend on sulfate-reduction activity, sulfide production, localized chemical gradients within biofilms, and metabolic interactions with other microorganisms, rather than their relative abundance alone [1]. Therefore, the low abundance or non-detection of SRB-associated taxa in this study should not be interpreted as evidence that SRB are unimportant in oral MIC. In the present dataset, *D. oralis*, an SRB-associated taxon [46], was detected only in one planktonic sample (S4; 0.12%; 1/13 participants) and was not detected in any biofilm sample. This sparse detection represents a limitation of the study. The participants were healthy adults, and samples were collected from orthodontic micro-implant-associated plaque, which may differ from more anaerobic oral niches, such as periodontal pockets, where SRB may be more readily enriched. In addition, low-abundance SRB may fall below the detection limit of V3–V4 16S rRNA gene amplicon sequencing, while ex vivo incubation may alter the composition and detectability of the original microbial communities. Future studies should employ targeted functional approaches, including dsrAB-targeted qPCR, metagenomic or metatranscriptomic sequencing, and enrichment-based assays, to determine the prevalence and activity of SRB in oral biofilms.

Our results implicate riboflavin-associated processes as a candidate factor associated with community-level corrosion behavior and a proposed interfacial redox mechanism that may involve EET. Riboflavin has been reported to act as an electron mediator in MIC systems; for example, exogenous riboflavin promoted pitting corrosion in *Desulfovibrio vulgaris* biofilms [29,47]. Flavin-associated electron transfer has also been linked to enhanced 316L SS corrosion in other microbial settings [25,28], and riboflavins are established diffusible electron shuttles in electroactive bacteria such as *Shewanella* [26,27,48]. In oral biofilm contexts, however, riboflavin-associated contributions have been comparatively less explored at the community level.

In our system, riboflavin was detected across all subject-derived consortia but remained undetectable in sterile controls, accumulated during incubation, and was highest in corrosion-active communities. Perturbation experiments provided experimental support for an association between riboflavin availability and corrosion: exogenous riboflavin increased *i*_corr_ and accelerated metal corrosion and dissolution, whereas roseoflavin reduced extracellular riboflavin availability and substantially decreased icorr and dissolved Cr/Fe concentrations. Crucially, these shifts in corrosion did not coincide with gross changes in biofilm biomass or surface-associated metabolic activity (crystal violet staining and surface ATP levels showed negligible differences), indicating that riboflavin availability modulates corrosion processes rather than globally altering growth. Nevertheless, roseoflavin is a riboflavin analogue that may exert broader biological effects, including interference with flavin-dependent metabolic processes beyond the reduction in extracellular riboflavin availability, and surface-associated ATP and biomass measurements alone cannot exclude more subtle changes in microbial physiology or community composition; the roseoflavin results should therefore be interpreted with caution. Moreover, in an assay without an added organic carbon source, the S3 community increased dissolved Cr and Fe release; riboflavin further enhanced, whereas roseoflavin decreased, metal release relative to untreated S3. Because the pH remained near neutral and did not differ appreciably from controls, these findings support a riboflavin-associated contribution to corrosion in this ex vivo model, consistent with a proposed interfacial redox process. While the data support an association between riboflavin availability and corrosion activity in our assay context, the precise micro-scale pathway(s) of electron transport at the biofilm–metal interface (e.g., direct shuttling vs. alternative redox coupling) remain to be defined by direct measurements of electron transfer and riboflavin exchange.

An important mechanistic distinction should be made between the corrosion pathway examined here and the metabolite-mediated pathway reported for *Streptococcus mutans*, an oral pathogen whose biofilms induce corrosion of 316L SS primarily through the production of acidic metabolites [23,49,50]. In the present study, the bulk pH of the incubation medium remained near neutral and did not differ appreciably from the sterile controls throughout the 7-day experiments (Figure A2). Under the buffered, oligotrophic conditions used here, bulk acidification is therefore unlikely to be the dominant driver of the observed corrosion. Instead, the corrosion-associated phenotype was consistently linked to extracellular riboflavin availability, and we propose that riboflavin acts as a diffusible redox mediator that can shuttle electrons between microbial cells and the steel surface [28], thereby promoting cathodic reactions and localized metal dissolution. Because direct measurements of electron flux and riboflavin exchange at the biofilm–metal interface were not performed, this mechanism is presented as a proposal consistent with the available data, and local interfacial pH or redox conditions within the biofilm may differ from the near-neutral bulk values. This mechanistic contrast with the acid-driven *S. mutans* pathway [23] highlights the specificity of the riboflavin-associated corrosion process examined here.

Reconstructed-community experiments provide insight into the community-level basis of the corrosion phenotype. The biofilm-enriched taxa selected for reconstruction, *C. tsuruhatensis*, *R. erythropolis*, and *T. aromatica*, showed high predicted pairwise metabolic complementarity in genome-informed analyses. Consistent with their potential involvement in electroactive biofilm phenotypes, *Comamonas* species have been detected in exoelectrogenic biofilms and bioelectrochemical systems [51], *Rhodococcus* strains have been reported to exhibit flavin-associated EET [52], and *Thauera* species have been isolated from electroactive anode biofilms [52,53]. Consistent with a potential cooperative community effect, *C. tsuruhatensis*, *R. erythropolis*, and *T. aromatica* monocultures formed sparse, discontinuous surface aggregates, whereas dual-species consortia displayed moderate surface coverage and limited three-dimensional maturation. In contrast, the *C. tsuruhatensis* + *R. erythropolis* + *T. aromatica* consortium formed a dense, continuous biofilm architecture and closely recapitulated the high-corrosion phenotype observed for the S3 consortium, including pitting severity.

Functionally, *C. tsuruhatensis* + *R. erythropolis* + *T. aromatica* induced markedly elevated *i*_corr_ and showed the deepest localized pits among the defined communities, with activity approaching that of the S3 consortium. Notably, *C. tsuruhatensis* alone did not reproduce the three-strain corrosion activity, and dual-species combinations partially restored it, suggesting that the high-corrosion outcome may depends on community interactions rather than individual taxa alone. Riboflavin-perturbation within the defined consortium further supported a proposed riboflavin-linked model: riboflavin supplementation promoted higher corrosion rate of 316L SS, whereas roseoflavin treatment reduced the measured corrosion phenotypes. Extracellular riboflavin accumulation in the defined system was observed primarily in *C. tsuruhatensis* and increased further in the presence of *R. erythropolis* and *T. aromatica*, whereas *R. erythropolis*, *T. aromatica*, and the *R. erythropolis* + *T. aromatica* combination showed minimal riboflavin levels. Importantly, riboflavin/roseoflavin effects on corrosion were not accompanied by parallel increases in overall biofilm biomass or surface-associated ATP, reinforcing the idea that riboflavin availability modulates corrosion-linked physiology in the interspecies context rather than broadly changing biomass.

This interpretation is consistent with the broader literature on multispecies biofilms, where community-level phenotypes often cannot be predicted from monoculture behavior [1]. Metabolic cross-feeding, spatial organization, competition, and metabolite sharing can alter biomass accumulation, local chemical gradients, and redox activity [54,55], and such interactions are increasingly considered important determinants of MIC dynamics [5,56]. In our system, the thicker and more continuous triple-species biofilm may have promoted retention of redox-active compounds and facilitated localized microenvironments that support corrosion. Nevertheless, direct measurements of riboflavin exchange kinetics and interspecies electron transfer at the interface were not performed; therefore, “riboflavin-linked interspecies interaction” should be regarded as a proposed mechanistic model supported by convergent evidence rather than directly visualized metabolite transfer.

The substantial variation in dissolved Cr and Fe release across the 13 subject-derived consortia may have clinical relevance. Corrosion products and surface degradation have been observed on retrieved orthodontic 316L SS components [57,58], yet clinical severity varies considerably among patients. Such variability has commonly been attributed to host and environmental factors such as oral hygiene, implantation duration, mechanical loading, saliva chemistry, and local inflammation [59]. Our findings indicate that, even under controlled ex vivo conditions, oral microbiota composition and predicted functional capacity may contribute to variation in corrosion activity. Specifically, communities combining taxa with predicted riboflavin-biosynthesis capacity with metabolically complementary partners may be ecologically primed to assemble corrosion-promoting biofilms. This does not imply that microbiota composition alone determines clinical failure; rather, it highlights the potential interaction between microbial ecology and physicochemical conditions that regulate passive-film stability. It should be emphasized, however, that these findings were obtained in a simplified ex vivo model, and direct extrapolation to clinical conditions is limited by the differences between the experimental system and the dynamic oral environment, including salivary flow and shear forces, mechanical loading, fluctuating oxygen levels, dietary exposure, pH variations, host immune factors, and long-term exposure conditions.

Several limitations should be considered. First, functional and metabolic inferences were based primarily on 16S rRNA gene sequencing, PICRUSt2 functional predictions, and genome-based MIP. These approaches estimate metabolic potential rather than directly measuring gene expression, metabolic flux, or interspecies metabolite exchange. Metatranscriptomic, metaproteomic, and targeted metabolomic analyses would help validate the proposed processes and identify which genes and metabolic routes are active in situ. Second, the three-strain consortium serves as a tractable model but necessarily simplifies the diverse oral microbiota, excluding low-abundance, uncultured, and host-derived contributors. In addition, SRBs, which are recognized contributors to MIC in many environments and can occur in oral niches, were not detected at appreciable relative abundance in the sequencing dataset of the subject-derived communities and were not included in the defined consortium. Nevertheless, it is long established that SRB cell numbers do not correlate with measured corrosion rates; accordingly, a low SRB abundance or even non-detection does not by itself allow their potential significance in the corrosion process to be dismissed. In addition, SRB are typically low-abundance anaerobes in oral niches, and their detection by 16S rRNA gene amplicon sequencing is constrained by the sampling procedure, sample transport and storage, DNA extraction efficiency, primer specificity, and sequencing depth; our single-time-point sampling of implant-associated plaque may therefore have underestimated their presence (Table A2). Dedicated approaches, such as targeted enrichment culture, quantitative PCR, and shotgun metagenomic sequencing, would be required to more definitively evaluate the presence and potential contribution of SRB in oral biofilm–metal systems. Their potential contribution to oral microbiota-associated corrosion therefore remains to be addressed in future studies. Third, roseoflavin, a riboflavin analogue used as a perturbation tool, may exert broader effects on flavin-dependent metabolism beyond reducing extracellular riboflavin availability, and the biomass and surface-ATP measurements performed here cannot exclude subtle changes in microbial physiology or community composition; the roseoflavin results were therefore interpreted as supporting associations rather than an established mechanism. Fourth, our ex vivo experiments were performed under static anaerobic conditions in simulated saliva, which do not fully reproduce the dynamic nature of the oral environment, including salivary flow and shear forces, mechanical loading, fluctuating oxygen levels, dietary exposure, pH variations, host immune factors, and long-term exposure conditions; the findings should therefore be regarded as evidence derived from an ex vivo experimental model rather than as direct evidence of clinical corrosion mechanisms. Fifth, the potentiodynamic polarization measurements represent endpoint snapshots obtained after 7 days of incubation rather than time-resolved corrosion monitoring; although these electrochemical results were corroborated by cumulative 7-day metal-release measurements, time-resolved techniques (e.g., electrochemical impedance spectroscopy or linear polarization resistance monitoring) would provide further insight into corrosion dynamics. Microbial abundance was not additionally monitored during the approximately 2-h electrochemical measurement window, and short-term community stability during polarization was therefore not independently verified. Finally, the cohort size (13 subjects) may not capture the full spectrum of oral microbiota diversity or corrosion phenotypes across broader populations. Sixth, the detailed multi-parameter characterization was performed for the S3 consortium selected on the basis of its highest Cr/Fe release in the primary screening; extending this characterization to additional consortia spanning the observed metal-release range (e.g., the next-highest- and lowest-release communities, S5 and S4) would further test the generalizability of the observed associations. Despite these limitations, the integration of donor-derived communities, interface-associated profiling, metabolite and electrochemical assays, and defined-community reconstruction provides an experimental ecological framework for studying biomaterial degradation as an emergent property of oral biofilm ecology.

## 5. Conclusions

This study shows that oral microbiota-associated corrosion of 316L SS varies with community composition and is consistent with a proposed role for species-dependent metabolic interactions. Subject-derived oral consortia accelerated metal release and localized surface corrosion, with substantial variation among communities. A defined reconstructed consortium comprising *C. tsuruhatensis*, *R. erythropolis*, and *T. aromatica* recapitulated the high-corrosion phenotype of the S3 consortium, including elevated *i*_corr_, extracellular riboflavin accumulation, biofilm formation, and induced pitting. Across both subject-derived and reconstructed systems, extracellular riboflavin levels were higher in corrosion-active communities and reached the highest levels in the reconstructed consortium. Riboflavin supplementation increased oral microbiota–mediated corrosion of 316L SS, whereas roseoflavin reduced extracellular riboflavin availabilityand was associated with lower corrosion-related measurements. These findings suggest that riboflavin is a functionally relevant metabolite associated with community interactions linked to corrosion at the oral biofilm–metal interface, and support an ecological framework for understanding microbiota-associated corrosion resistance of oral biomaterials.

## Data Availability

The data presented in this study are available on request from the corresponding author due to restrictions related to participant privacy and informed consent, which do not permit full public sharing of the raw dataset.

## Abbreviations

The following abbreviations are used in this manuscript:

MIC	Microbiologically influenced corrosion
OTU	Operational taxonomic unit

## Appendix A

**Table A1:** Clinical characteristics of participants.

ID	Group	Sex	Age
S1	Healthy	M	20
S2	Healthy	F	23
S3	Healthy	M	22
S4	Healthy	F	22
S5	Healthy	M	23
S6	Healthy	F	24
S7	Healthy	M	21
S8	Healthy	F	27
S9	Healthy	M	23
S10	Healthy	F	25
S11	Healthy	M	28
S12	Healthy	F	23
S13	Healthy	M	25

**Table A2:** Subject-level detection and relative abundance of *D. oralis* in planktonic and biofilm communities, and its correlation with metal release.

Subject/Summary	*D. oralis* in Planktonic Community	*D. oralis* in Biofilm Community
S1	0	0
S2	0	0
S3	0	0
S4	0.12%	0
S5	0	0
S6	0	0
S7	0	0
S8	0	0
S9	0	0
S10	0	0
S11	0	0
S12	0	0
S13	0	0
Detected subjects, n/N (%)	1/13 (7.7%)	0/13 (0%)
Relative abundance among positive samples	0.12%	—
Spearman’s *r* versus Cr release	−0.46	—
Spearman’s *r* versus Fe release	−0.46	—

Table note: Values represent relative abundance determined by 16S rRNA gene amplicon sequencing. Zero indicates that *D. oralis* was not detected in the corresponding sample. *D. oralis* was detected only in the planktonic community of subject S4 (0.12%) and was not detected in any biofilm sample. Correlation coefficients should be interpreted with extreme caution because only one of 13 subjects showed detectable *D. oralis*, resulting in insufficient variation for a robust association analysis.
